# Editorial: Radioimmunotherapy—Translational Opportunities and Challenges

**DOI:** 10.3389/fonc.2020.00190

**Published:** 2020-02-21

**Authors:** Udo S. Gaipl, Gabriele Multhoff, A. Graham Pockley, Franz Rödel

**Affiliations:** ^1^Department of Radiation Oncology, Friedrich-Alexander-Universität Erlangen-Nürnberg, Universitätsklinikum Erlangen, Erlangen, Germany; ^2^Radiation Immuno-Oncology Group, Center of Translational Cancer Research (TranslaTUM), Klinikum rechts der Isar, Technical University Munich (TUM), Munich, Germany; ^3^John van Geest Cancer Research Centre, Nottingham Trent University, Nottingham, United Kingdom; ^4^Department of Radiotherapy and Oncology, University of Frankfurt, Frankfurt, Germany

**Keywords:** translational reseach, immunotherapy, radiotherapy, radioimmunotherapy, vaccination, tumor stroma, immune checkpoint inhibitors

It has become evident that radiotherapy has both, immune suppressive, and immune activating properties (1). This is why this important component of cancer treatment should be combined with immune therapies to shift the balance toward immune activation against tumor cells. During the last decade a manifold of pre-clinical work was put into investigation of combination of radiotherapy either with additional immune stimulants such as cytokines or vaccines or in combination with antibodies that target immune suppressive molecules such as immune checkpoint inhibitors. Luckily, some of these approaches are currently tested in clinical trials, high lightening the huge translational opportunities by examination of modes of action of radiotherapy in combination with immunotherapy; named in this special issue *radioimmunotherapy*. However, one has always to keep in mind that many challenges do still exist such as what is the best sequence and timing of joint applications, what are the best immunotherapy approaches, how to overcome tumor resistances, what about healthy tissue cytotoxicity, or which biomarkers or matrices of biomarkers are most beneficial for patients stratification, just to mention the most burning ones. The articles in this special issue grab many of these challenges.

Integration of radiotherapy in multimodal tumor treatments is not to be challenged since above half of the tumor patients do receive it during their diseases history. Further, it has been proven that locally applied radiotherapy does not destroy the immune system in a way that additional immunotherapy is not feasible. Voos et al. show that exposing cells of the adaptive immune system, namely T cells, to radiation even results in their Ca2^+^-dependent activation. Furthermore, radiation-exposed T cells adhered better to endothelial cells (Voos et al.). Nevertheless, these features might impact both, toxic effects of radiation and a better T cell-mediated treatment response. The latter can be enhanced by immune activatory cytokines. This is the focus of the work of Palata et al. who review on the efficacy of combination treatments of radiotherapy with IL-2, IFN-alpha, TNF-alpha, GM-CSF, and immunocytokine-based approaches which are already tested in clinical trials. Additionally, work about IL-12 and IL-15-based immunotherapy approaches is presented. This again high lights the huge translational opportunities of radioimmunotherapies (Palata et al.). Besides cytokines, active stimulation of the immune system can be achieved by vaccination approaches. Seitz et al. demonstrate for the first time in pre-clinical model systems that radiotherapy can be combined with vaccination with syngeneic whole-tumor cell vaccine generated by high hydrostatic pressure, mimicking in cancer patients autologous vaccine from their own tumor cells. Radiotherapy thereby acts as an adjuvant for the vaccine that contains many tumor-associated antigens (adjuvanticity plus antigenicity) (Seitz et al.). The work of Liu et al. gives some additional hints that whole-body irradiation with low doses enhances the *in situ* vaccine effects of locally applied radiotherapy. This reflects the complexity and diversity of mechanisms of radiation-induced immune modulation. While low radiation doses mostly seem to enhance immune cell infiltration into tumors, higher doses do induce immunogenic cancer cell death, and create an immune stimulatory micro-environment for the attracted immune cells.

The change in the stromal compartments of tumors following radiation exposure have to be followed very detailed for future radioimmunotherapy optimization. Martinez-Zubiaurre et al. particularly summarize the time-dependence of stromal changes following radiation exposure. Only short windows of opportunities might exist for effective combination of radiotherapy with immune therapies (Martinez-Zubiaurre et al.). Sevenich summarizes the key features how to turn immunological “cold” into “hot” tumors and discusses an additional challenging fact about immune properties of different tumor entities. Particularly brain tumors have highly immune suppressive properties and are located at an immune privileged site. Nevertheless, immune cells do infiltrate brain tumors and distinct well-elaborated combinations of radiotherapy with immune therapy could be successful for primary and metastatic brain tumors (Sevenich). Buchwald et al. review about pre-clinical and clinical work dealing with radioimmunotherapy-induced immune responses against the primary, irradiated, and abscopal, non-irradiated, tumor masses. They stress that besides the tumor location, timing, dose, and fractionation strongly impacts anti-tumor immune responses. They focus on T cell exhaustion and on how radiotherapy should be combined with immune checkpoint-inhibitors such as antibodies targeting the PD-1/PD-L1 network in this context (Buchwald et al.). One has never to forget that classical tumor features such as hypoxic regions have to be taken into account, as these regions do also show immune suppressive features such as increased amounts of regulatory T cells and myeloid-derived suppressor cells and increased concentrations of TGF-beta. Eckert et al. stress that particularly patients with hypoxic tumors might therefore benefit from radioimmunotherapies.

Since multiple immune suppressive properties of tumors do exist, combined approaches that aim to both, activate tumor-reactive T cells, and neutralize exhausted T cells should be more efficient. Ostrand-Rosenberg et al. report about bispecific T cell engagers (BiTE) that activate and target cytotoxic T cells and natural killer T cells to kill PD-L1 expressing tumor cells. They further stress that additional combination with co-stimulatory sCD80 increases T cell-mediated anti-tumor immune responses and should be tested in the future in combination with radiotherapy (Ostrand-Rosenberg et al.). Another innovative approach of targeted stimulation of anti-tumor immune responses is the use of functionalized superparamagnetic iron oxide nanoparticles (SPIONs), as outlined by Janko et al. These nanoparticles have the great advantage to locally targeting the tumor by reducing side effects. Besides cytotoxic agents, immune modulatory molecules can be coupled to these particles and by application of an external mantic field, additional heating of the tumor is possible, again contributing to enhanced immunogenic features of the tumor (Janko et al.).

In all of the described approaches of combining radiotherapy with immune modulators, patient's stratification is of key importance. Here, immune contextures play a central role, besides genetic features of the tumor (e.g., tumor mutational burden) and viral pathogenic factors, since the latter seem to impact radiation sensitivity and antitumor immunity (2). Clinical data about association of viral polyomavirus load and CD8^+^ T cell infiltration into Merkel cell carcinoma are presented by von der Grün et al. While high viral load was associated with worse overall survival (OS), high intratumoral CD8^+^ T cell was associated with improved OS. Importantly, expression of immune suppressive PD-L1 was correlated with increased T cell infiltration. These clinical observations once more stress that multiple immune features do impact on efficient anti-tumor immune responses.

Radiotherapy in this context has functions as immune stimulator, immune suppressor, and as fine-tuner of immune responses. Let's go ahead with multimodal radioimmunotherapies for cancer. The knowledge about joint actions of radiotherapy and immunotherapy is increasing daily and the results of the ongoing clinical trials will help to further improve personalized radioimmunotherapies.

## Author Contributions

All authors listed have made a substantial, direct and intellectual contribution to the work, and approved it for publication.

### Conflict of Interest

The authors declare that the research was conducted in the absence of any commercial or financial relationships that could be construed as a potential conflict of interest.
